# The Cell Wall-Derived Xyloglucan Is a New DAMP Triggering Plant Immunity in *Vitis vinifera* and *Arabidopsis thaliana*

**DOI:** 10.3389/fpls.2018.01725

**Published:** 2018-11-28

**Authors:** Justine Claverie, Suzanne Balacey, Christelle Lemaître-Guillier, Daphnée Brulé, Annick Chiltz, Lucie Granet, Elodie Noirot, Xavier Daire, Benoît Darblade, Marie-Claire Héloir, Benoit Poinssot

**Affiliations:** ^1^Agroécologie, Agrosup Dijon, INRA, Université Bourgogne Franche-Comté, CNRS ERL, Dijon, France; ^2^Elicityl, Crolles, France

**Keywords:** DAMP-triggered immunity, induced resistance, mitogen-activated protein kinase, *Botrytis cinerea*, elicitor

## Abstract

Damage-associated molecular patterns (DAMPs) are endogenous molecules that can activate the plant innate immunity. DAMPs can derive from the plant cell wall, which is composed of a complex mixture of cellulose, hemicellulose, and pectin polysaccharides. Fragments of pectin, called oligogalacturonides (OG), can be released after wounding or by pathogen-encoded cell wall degrading enzymes (CWDEs) such as polygalacturonases (PGs). OG are known to induce innate immune responses, including the activation of mitogen-activated protein kinases (MAPKs), production of H_2_O_2_, defense gene activation, and callose deposition. Thus, we hypothesized that xyloglucans (Xh), derived from the plant cell wall hemicellulose, could also act as an endogenous elicitor and trigger a signaling cascade similar to OG. Our results indicate that purified Xh elicit MAPK activation and immune gene expression in grapevine (*Vitis vinifera*) and Arabidopsis (*Arabidopsis thaliana*) to trigger induced resistance against necrotrophic (*Botrytis cinerea*) or biotrophic (*Hyaloperonospora arabidopsidis*) pathogens. Xh also induce resveratrol production in grapevine cell suspension and callose deposition in Arabidopsis which depends on the callose synthase PMR4. In addition, we characterized some signaling components of Xh-induced immunity using Arabidopsis mutants. Our data suggest that Xh-induced resistance against *B. cinerea* is dependent on the phytoalexin, salicylate, jasmonate, and ethylene pathways.

## Introduction

Plant resistance is based on their ability to perceive microorganisms and induce immune responses to stop their invasion. This recognition is possible *via* the perception of eliciting molecules released during the plant/pathogen interaction. These elicitors, called microbe-associated molecular patterns (MAMPs) or pathogen-associated molecular patterns (PAMPs), comprise conserved molecular patterns such as peptides of bacterial flagellin (flg22) or fungal chitin oligomers (Newman et al., 2013; Brulé et al., 2018) and activate a set of defense-associated responses termed PAMP-triggered immunity (PTI; Jones and Dangl, 2006). Plants are also able to distinguish the non-self from the damaged-self *via* a complex recognition system (Trdá et al., 2015). Thus, endogenous elicitors released from the wounded host have been described as damage-associated molecular patterns (DAMPs) including extracellular ATP, peptides, or fragments of cell walls (Heil and Land, 2014). Plant cell walls are composed of a complex mixture of polysaccharides, such as cellulose, hemicellulose, and pectin. Pectin-derived oligogalacturonides (OG) are well-characterized DAMPs as they elicit a broad range of defense responses in several plant species (Ferrari et al., 2013). In the model plant *Arabidopsis thaliana* (Arabidopsis), OG trigger the phosphorylation of two mitogen-activated protein kinases (MAPKs), called *At*MPK3 and *At*MPK6 (Denoux et al., 2008). *At*MPK3 is involved in the basal resistance against *Botrytis cinerea* whereas *At*MPK6 is essential for the OG- or flg22-induced resistance against this pathogen (Galletti et al., 2011). Moreover, OG induce a nitric oxide production (Rasul et al., 2012) and an oxidative burst mediated by the NADPH oxidase Respiratory Burst Oxidase Homolog D (AtRbohD; Rasul et al., 2012). Activation of these signaling components leads to a defense transcriptome reprogramming including the transcription of *Phytoalexin Deficient 3* (*PAD3;* Galletti et al., 2008) and *Pathogenesis Related 1* (*PR-1*; Manzoor et al., 2013). Later, a callose production leads to a cell wall reinforcement dependent on the callose synthase Powdery Mildew Resistant 4 (PMR4; Ferrari et al., 2013) and AtRbohD (Galletti et al., 2008). Last, OG are able to trigger resistance of *A. thaliana* and grapevine against *B. cinerea* (Aziz et al., 2004; Ferrari et al., 2007), protection of wheat against powdery mildew (Randoux et al., 2009), and potato against *Pectobacterium carotovorum* (Davidsson et al., 2013). Interestingly, the expression of a fungal PG coupled to a polygalacturonase-inhibiting protein (PGIP) in transgenic Arabidopsis demonstrates that the *in vivo* release of OG is sufficient to trigger the plant immunity (Benedetti et al., 2015). The mechanism of OG perception was characterized in Arabidopsis by the identification of the wall-associated kinase (WAK) family. First, both WAK1 and WAK2 ectodomains have been shown to bind pectin *in vitro* (Kohorn et al., 2009), then WAK1 has been hypothesized to act as a receptor of OG (Decreux and Messiaen, 2005; Cabrera et al., 2008). WAK1 is composed of an extracellular domain containing epidermal growth factor motifs, a transmembrane domain, and an intracellular Ser/Thr kinase domain (Ferrari et al., 2013). Finally, a chimeric receptor approach revealed that WAK1 acts *in vivo* as a receptor of OG (Brutus et al., 2010).

Besides OG, other plant cell wall compounds, such as the cellulose-derived oligomers of cellobiose and cellodextrins, act as DAMP and trigger defense-like responses in Arabidopsis and grapevine, respectively (Aziz et al., 2007; Souza et al., 2017). Results indicate that cellobiose triggers a signaling cascade that shares some similar responses with OG. Thus, cellobiose treatment leads to the activation of MAPKs, an early and transient calcium variation and the expression of the defense-related gene *WRKY30* (Souza et al., 2017). Global Arabidopsis transcriptome profiles are very similar after cellobiose or OG treatment. However, cellobiose does not stimulate reactive oxygen species (ROS) production or callose deposition (Souza et al., 2017). At the opposite, cellodextrins (linear β-1,4 glucans) elicit ROS production and the expression of grapevine defense-related genes leading to induced resistance against *B. cinerea* (Aziz et al., 2007).

Xyloglucan oligosaccharides (Xh) are the major components of hemicellulose contained in the cell wall of (dicotyledonous) plants. Previous studies indicated that Xh may play a role in the regulation of plant growth and development in different plant species (Fry et al., 1993; Vargas-Rechia et al., 1998). Indeed, treatment with Xh of tobacco cell cultures and whole plants results in accelerated cell elongation and division, changes in primary root growth, and upregulation of defense-related gene expression (González-Pérez et al., 2014). Moreover, Xh can increase plant resistance to abiotic stress, in particular cold or water stress, when used at low concentrations (Salvador and Lasserre, 2010). The aim of this study was to evaluate the efficacy of Xh to trigger the grapevine (*Vitis vinifera*) immunity and to decipher its mode of action in the model plant *A. thaliana*. First, eliciting properties of Xh were investigated in grapevine cells by analyzing some immune responses and efficacy of Xh to induce resistance was evaluated in the grapevine/*B. cinerea* interaction. Then, Arabidopsis plants were used to characterize some signaling components involved in the Xh-induced immunity. In this study, OG were used as a positive control.

## Results

### Quality Control of the Purified Xyloglucans

**FIGURE 1:**
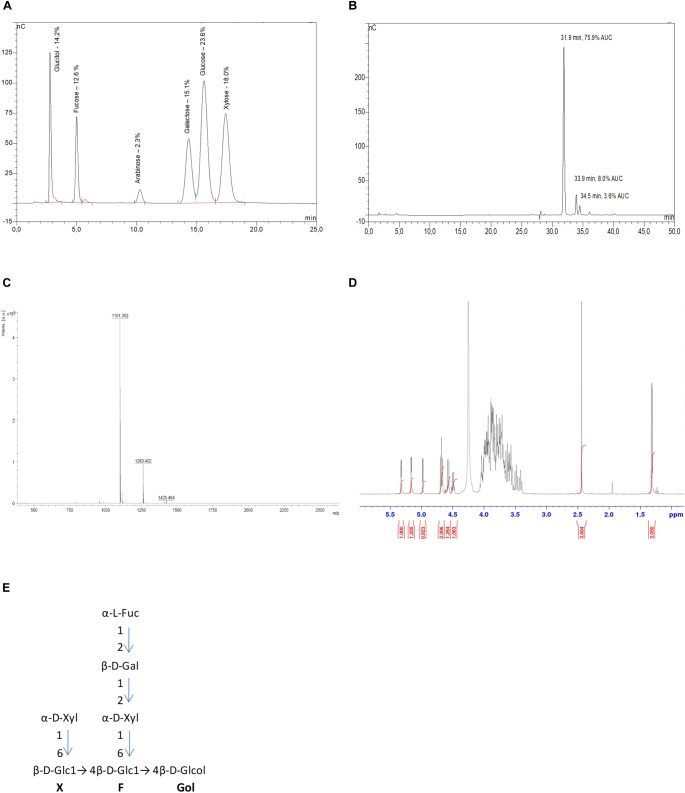
Xyloglucan (Xh) characterization. **(A)** HPAEC-PAD PA1 chromatogram of hydrolyzed Xh. Xh monosaccharide composition is based on glucitol, fucose, arabinose, galactose, glucose, and xylose. The specific identification of monosaccharides is based on internal standards. Indicated percentages correspond to dry weight of each monosaccharide when 100 mg of Xh are injected, based on calibration curves for each standard. **(B)** HPAEC-PAD PA100 chromatogram of native Xh. The main peak (75.9% AUC) at 31.9 min corresponds to heptamaloxyloglucan DP7 XFGol and the two minor peaks at 33.9 and 34.5 min correspond to oligomers of DP8 and DP9, respectively. No free monosaccharides are detected in Xh. **(C)** Xh MALDI-TOF spectrum (ES+): heptamaloxyloglucan (DP7, XFGol) MS (ES+): *m/z* = 1101 [M+Na]+, DP8 (LFGol) MS (ES+): *m/z* = 1263 [M+Na]+, DP9 (GLFGol) *m/z* = 1425 [M+Na]+. **(D)** 1H-NMR spectrum of Xh. 1H-NMR (400 MHz, D2O). 5.31 (d, 1H, H-1Fuc), 5.15 (d, 1H, H-1Xyl’), 4.98 (d, 1H, H-1Xyl”), 4.68 (dd, 2H, H-1Glc’,H-1Glc”), 4.58 (d, 1H, H-1Gal), 4.5 (d, 1H, H-5Fuc), 2.42 (m, 4H, internal standard: ISTD), 1.30 (d, 3H, 3.05, fucose methyl). **(E)** Deduced structure of the main purified xyloglucan (heptamaloxyloglucan or Xh DP7) presented according to the oligosaccharide nomenclature described by Fry et al. (1993). Structures of the minor components are presented in Supplementary Figure S1.

The Xh used in this study are highly purified xyloglucan oligomers obtained by enzymatic extraction and purification from apple pomace. To perform a detailed characterization of Xh, three complementary analytical methods have been used: high-pressure anion exchange chromatography coupled to pulsed amperometry detection (HPAEC-PAD), matrix-assisted laser desorption-ionization time-of-flight (MALDI-TOF) mass spectrometry, and proton nuclear magnetic resonance spectroscopy (^1^H-NMR). Because the separation of the α- and β-anomers of reducing oligosaccharides leads to complicated chromatograms and difficult interpretations in ^1^H-NMR analysis, xyloglucans have been reduced by NaBH_4_ at the first step of the process. This explains the existence of glucitol (sorbitol) in the chromatogram realized after the acid hydrolysis of the Xh by trichlorofluoroacetic acid (Figure 1A). Thus, determination of monosaccharide composition performed by HPAEC-PAD on PA1 column showed that the extract composition is based on glucitol [14.2% of dry weight (DW)], fucose (12.6% DW), arabinose (2.3% DW), galactose (15.1% DW), glucose (23.6% DW), and xylose (18.0% DW) monosaccharides (Figure 1A). The different monosaccharides obtained are corresponding to the previously described apple xyloglucan composition (Watt et al., 1999), except that traces of arabinose have been more described for arabinose-containing xyloglucans in sycamore (Kiefer et al., 1990) or different monocotyledons (Hsieh and Harris, 2009). The separation of the purified Xh oligomers realized by HPAEC-PAD PA100 revealed that the main peak on the chromatogram [31.9 min, 75.9% of the area under the curve (AUC), Figure 1B] was associated with the main compound detected by mass spectrometry (*m/z* = 1101 [M+Na^+^], Figure 1C). This mass is consistent with a xyloglucan oligomer structure with a degree of polymerization of 7 (DP 7). The other two minor peaks (at 33.9 min, 8.0% AUC and 34.5 min, 3.6% AUC; Figure 1B) were associated to the two compounds detected by mass spectrometry with *m/z* = 1263 and 1425 (Figure 1C), which correspond to xyloglucan oligomers of DP8 and DP9, respectively. This characterization was supported by the complementary analysis of the ^1^H-NMR spectrum (Figure 1D) that enabled the identification of heptamaloxyloglucan or XFGol (according to xyloglucan nomenclature described by Fry et al., 1993), as the main component of the extract. Heptamaloxyloglucan (Xh DP7) has a backbone of three ß1,4-linked glucose residues. Glucoses Glc’ and Glc” are substituted with α1,6-linked xylose sidechains and the xylose’ is capped by a galactose residue followed by a fucose residue (Figures 1D,E). Moreover, the relative amount of each monosaccharide determined by HPAEC-PAD on PA1 column (Figure 1A) is consistent with the expected ratio in the heptamaloxyloglucan (Supplementary Table S1). The two other minor components have been identified as xyloglucan oligomers LFGol (DP8) and GLFGol (DP9; Supplementary Figure S1). The ^1^H-NMR signal for arabinose was too low to determine precisely its location in a putative alternative oligomer that might be present at very low level in Xh. Of importance, the absence of galacturonic acid detected by these different analytical approaches supports that Xh are free of OG. Similarly, the HPAEC-PAD PA100 realized in the same conditions confirmed that a cellobiose standard possesses a different retention time (11.86 min) than Xh oligomers (Supplementary Figure S1C). So these analyses excluded a putative contamination of the purified Xh by OG or cellobiose.

### Xyloglucan-Triggered Immune Responses in Grapevine

**FIGURE 2:**
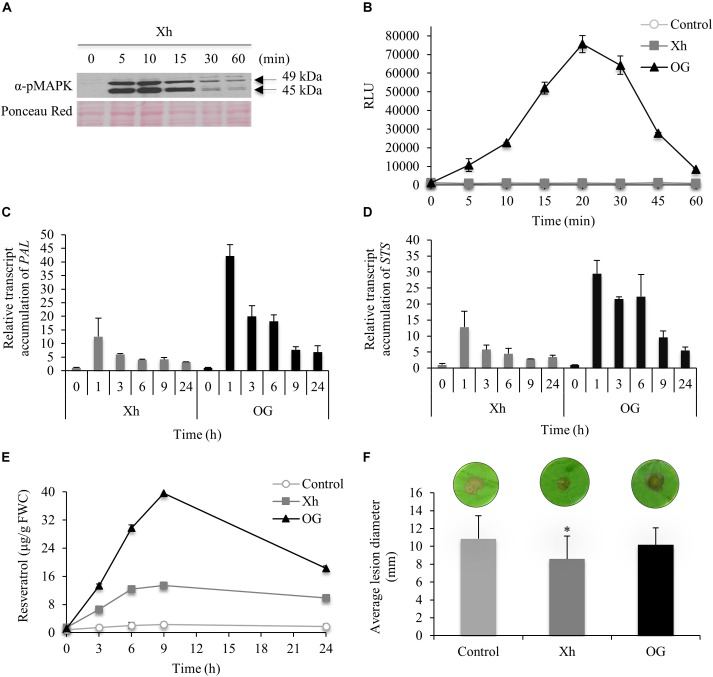
Immune responses triggered by Xh in grapevine (*Vitis vinifera*) cells and Xh-induced resistance in grapevine leaf disks. **(A)** Phosphorylation of two mitogen-activated protein kinases (MAPKs) in grapevine cells treated by Xh (1 mg/ml) detected by immunoblotting with α-pERK1/2. Results are from one representative experiment out of three. **(B)** H_2_O_2_ production in grapevine cells treated with Xh or OG (1 mg/ml) detected by chemiluminescence using a luminol-peroxidase-based assay. Data represent mean ± SD of one representative experiment out of three. Relative expression kinetics of two defense-related genes encoding for phenylalanine ammonia lyase (*PAL*; **C**) and stillbene synthase (*STS*; **D**) in grapevine cells treated with Xh or OG (1 mg/ml). Results represent the mean ± SD of three biological replicates and the transcript levels were arbitrary set as 1 for time 0. **(E)** Resveratrol quantification in the culture medium of grapevine cells treated with Xh or OG (1 mg/ml). Data represent mean of three independent experiments ± SD. **(F)** Xh-induced resistance against *Botrytis cinerea*. Leaf disks were incubated for 48 h on aqueous solutions containing Xh or OG (5 mg/ml) before inoculation with conidial suspension (5.10^4^ conidia/ml) of *B. cinerea* (40 disks per condition). Disease assessment was determined measuring the average diameter of lesions formed 3 days post inoculation (dpi). Data represent the average diameter of lesions ± SD of three independent experiments. Asterisks indicate significantly different values between treated vs control treatments according to Student’s *t*-test (*P* < 0.05). A representative leaf disk for each treatment is shown.

As MAPKs play a key role in innate immune signaling, their immunodetection in grapevine cells treated with Xh was analyzed using an antibody raised against a conserved phosphorylated peptide contained in the human active MAPKs. Xh treatment induced a dose-dependent MAPK phosphorylation in grapevine cell suspensions, with a stronger signal at 1 mg/ml (Supplementary Figure S2A). The xyloglucan concentration of 1 mg/ml was then used to study the defense-related events in grapevine. The time course of activation revealed a rapid and transient phosphorylation of two MAPKs with relative molecular masses of 49 and 45 kDa, respectively (Figure 2A). Their activation was detected within 5 min of treatment, peaked at 10 min and then decreased after 15 min.

Another early defense response is the generation of ROS. Using a luminol-peroxidase-based assay to quantify hydrogen peroxide, we investigated whether grapevine cells were able to produce H_2_O_2_ in response to Xh treatment. As previously shown by Aziz et al. (2004), the OG-mediated oxidative burst was strongly induced compared to control treatment (Figure 2B). Conversely, no H_2_O_2_ production was detected in Xh-treated grapevine cell suspensions (Figure 2B).

Phenylalanine ammonia lyase (PAL) is a key enzyme of the phenylpropanoid pathway. Downstream to PAL, stilbene synthase (STS) is responsible for the last step of resveratrol biosynthesis, the main phytoalexin produced in grapevine (Coutos-Thevenot et al., 2001). In OG-elicited cells, *PAL* and *STS* transcript accumulation was detected after 1 h of treatment (42- and 29-fold increase, respectively) before decreasing until the end of the experiment (Figures 2C,D). In Xh-treated cells, *PAL* and *STS* transcripts were also detected after 1 h of treatment, reaching a 12- and 13-fold accumulation, respectively, before slowly declining (Figures 2C,D). The resveratrol accumulated in the extracellular medium, peaked at 9 h after Xh and OG treatments [13 and 40 μg/g fresh weight of cells (FWC), respectively; Figure 2E]. The amount of resveratrol was constant and very low in control cells (≤2 μg/g FWC).

To know if the immune responses triggered by Xh lead to resistance, the efficacy of Xh-induced resistance was also investigated. Grapevine leaf disks were treated with Xh or OG for 48 h prior to inoculation with the necrotrophic fungus *B. cinerea*. At 3 days post inoculation (dpi), Xh treatment triggered a significant induced resistance against *B. cinerea* as it reduced by about 20% the average lesion diameter compared to control plants (Figure 2F). On the other hand, OG treatment did not induce a significant resistance against *B. cinerea* on grapevine leaf disks. Furthermore, Xh did not significantly modify the growth of *B. cinerea in vitro* (Supplementary Figure S3), excluding a putative direct toxic effect on the conidia germination or the mycelial growth. To confirm that the elicitor activity was linked to the oligomer structure of xyloglucans, an acid hydrolysis of Xh was performed then the induced resistance against *B. cinerea* was tested. Our results indicated that the hydrolyzed Xh (Xh_H) lost their resistance inducing activity confirming that their oligomer structures are necessary to trigger plant immunity (Supplementary Figure S4A).

### Xh Elicit MPK3 and MPK6 Phosphorylation, Immune Gene Expression, and Resistance Against *B. cinerea* in Arabidopsis

**FIGURE 3:**
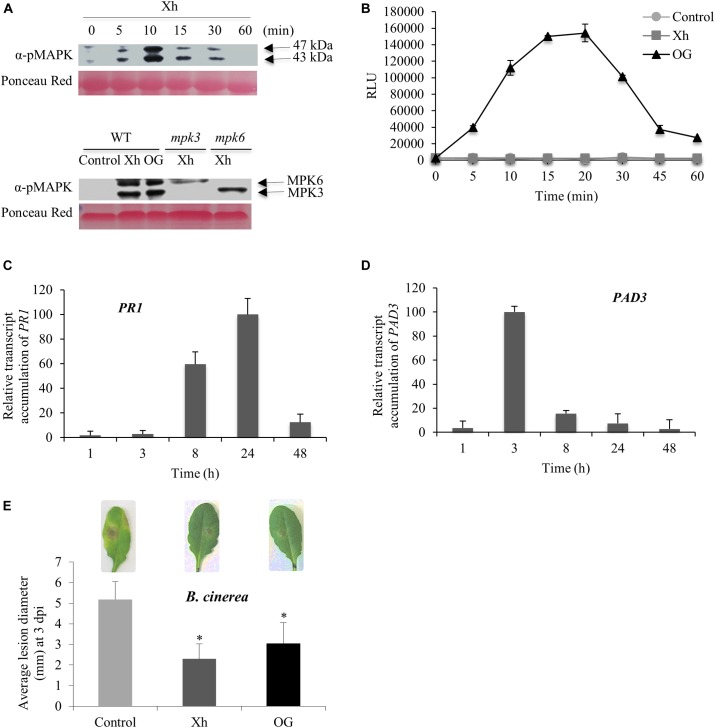
Immune responses and resistance against *B. cinerea* induced by Xh or OG in Arabidopsis. **(A)** Upper lane: phosphorylation of two mitogen-activated protein kinases (MAPKs) in Col-0 plants treated by Xh (1 mg/ml) detected by immunoblotting with α-pERK1/2. Lower lane: phosphorylation of two mitogen-activated protein kinases (MAPKs) in Col-0, *mpk3* and *mpk6* mutant plants treated by Xh (1 mg/ml) and detected at 10 min by immunoblotting with α-pERK1/2. Results are from one representative experiment out of three. **(B)** H_2_O_2_ production in Arabidopsis cells treated with water (control), Xh, or OG (1 mg/ml) detected by chemiluminescence using a luminol-peroxidase-based assay. Data represent means ± SD from one representative experiment out of three. **(C,D)** Time course of relative expression of defense genes encoding pathogenesis-related protein 1 (*PR1*) and phytoalexin deficient 3 (*PAD3*) after Xh treatment measured by qPCR. Results represent the mean ± SD of three biological replicates. Data were expressed as percentage of the Xh-treated Col-0, the strongest time point induction set as 100%. **(E)** Xh-induced resistance against *B. cinerea* in Arabidopsis. Two days after treatment by spraying with water (control), Xh (2.5 mg/ml), or OG (2.5 mg/ml), Col-0 plants were inoculated with *B. cinerea* (5.10^4^ conidia ml^-1^) and disease symptoms were measured at 3 dpi. Results represent the mean lesion diameter ± SD of four independent experiments. Asterisks indicate statistically significant differences between control and elicitor-treated plants using a Student’s *t*-test (*P* < 0.05). A representative leaf for each treatment is shown.

To further investigate the mode of action of Xh, we performed a similar dose-dependent MAPK phosphorylation assay in *A. thaliana* (Supplementary Figure S2B). Also in this case a strong phosphorylation signal was obtained for a Xh concentration between 0.5 and 1 mg/ml (Supplementary Figure S2B). The concentration of 1 mg/ml was thereafter used to study the defense-related events in Arabidopsis (Figure 3). Phosphorylation of two MAPKs with relative molecular masses of 47 and 43 kDa, respectively, was visible at early time points, with a maximum at 10 min after Xh treatment (upper lane in Figure 3A).

In order to identify the two MAPKs phosphorylated in response to Xh, we used *Arabidopsis mpk3* and *mpk6* mutants, respectively, deleted in MPK3 or MPK6. Figure 3A (lower lane) indicated that the two MAPKs phosphorylated in response to Xh are MPK3 and MPK6 as one immuno-detected band disappeared in each corresponding mutant *mpk3* or *mpk6*.

As previously observed in grapevine, no H_2_O_2_ signal was detected in response to Xh in Arabidopsis cell suspensions (Figure 3B). Nevertheless, our results confirmed that OG induce an oxidative burst in Arabidopsis (Figure 3B), as described previously (Galletti et al., 2008; Dubreuil-Maurizi et al., 2011; Rasul et al., 2012).

Elicited plants undergo transcriptional reprogramming leading to defense-related gene expression such as *Pathogenesis-Related protein 1* (*PR1*) and *PAD3*, *PR1* being often considered as a SA marker gene (Maleck et al., 2000). *PAD3* encodes the cytochrome P450 CYP71B15 enzyme which catalyzes the last step of the biosynthesis of the Arabidopsis phytoalexin camalexin (Schuhegger et al., 2006). As shown in Figures 3C,D, our data indicate that Xh treatment resulted in a high accumulation of *PR1* transcripts at 8 and 24 h post-treatment (hpt) whereas *PAD3* transcripts accumulated at 3 and 8 hpt.

To investigate the efficacy of Xh in preventing plant infection, protection assays against the necrotrophic fungus *B. cinerea* have been performed on Arabidopsis plants. As previously described by Ferrari et al. (2007), OG treatment decreased by ∼40% the *B. cinerea* lesion diameter at 3 dpi (Figure 3E). Interestingly, Xh treatment also strongly reduced the *B. cinerea* lesion diameter by ∼50% at 3 dpi (Figure 3E). As expected, the hydrolyzed Xh (Xh_H) was also unable to trigger the induced resistance against *B. cinerea* in Arabidopsis (Supplementary Figure S4B).

### Xh Trigger a PMR4-Dependent Callose Deposition in Arabidopsis and Induce the Overexpression of Defense Genes During *B. cinerea* Infection

**FIGURE 4:**
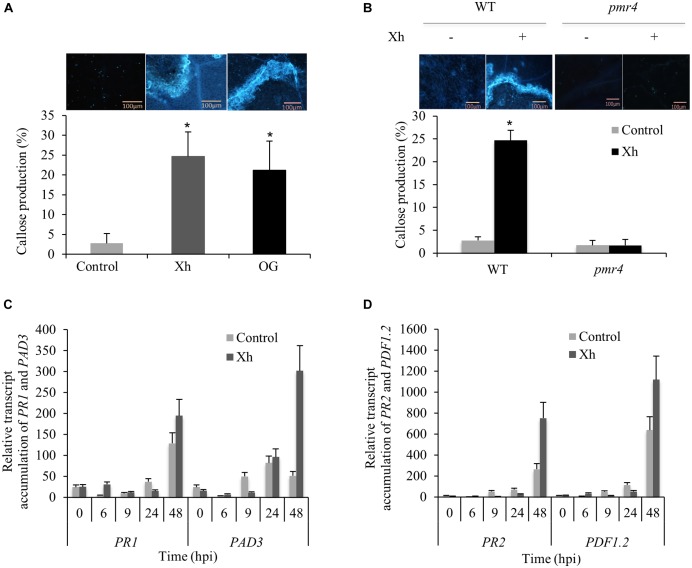
Xh trigger a PMR4-dependent callose deposition in Arabidopsis and the overexpression of defense genes during *B. cinerea* infection. **(A)** Callose quantification at 3 dpi detected by aniline blue staining in Arabidopsis Col-0 plants treated with water (control), Xh, or OG (2.5 mg/ml). **(B)** Callose quantification at 3 dpi detected by aniline blue staining in WT (Col-0) and *pmr4* mutant treated with water (–) or Xh (+; 2.5 mg/ml). Values represent means ± SD of at least 10 observed fields. A representative fluorescence microscopy imaging for each treatment is shown. Bar: 100 μm. Asterisks indicate statistically significant differences between control and elicitor-treated plants using a Student’s *t*-test (*P* < 0.05). Time course of relative expression of defense genes encoding pathogenesis-related protein 1 (*PR1*; **C**), phytoalexin deficient 3 (*PAD3*; **C**), pathogenesis-related protein 2 (*PR2*; **D**), and plant defensin 1.2 (*PDF1.2*; **D**) during Xh-induced resistance against *B. cinerea* measured by qPCR. Results represent the mean ± SD of three biological replicates after normalization by the housekeeping gene *At4g26410* (Czechowski et al., 2005). Hpi, hours post inoculation.

To determine if the Xh treatment triggers callose deposition, Arabidopsis plants were sprayed with Xh or OG 2 days before inoculation with *B. cinerea* and stained with aniline blue for callose visualization at 3 dpi. Compared to control, Xh- and OG-treated plants showed a significant increase of callose deposition 3 days after *B. cinerea* inoculation (Figure 4A).

During plant pathogen response, *PMR4* encodes the callose synthase responsible for the deposition of callose in papillae (Nishimura et al., 2003). Compared to WT Col-0 plants, Xh treatment in the *pmr4* mutant resulted in a dramatic decrease of callose deposition indicating that Xh-triggered callose production is dependent on PMR4 (Figure 4B).

As we demonstrated that Xh treatment directly elicits the expression of defense genes such as *PR1* and *PAD3* in Arabidopsis, we investigated if immune genes were also induced in Xh-treated plants challenged with *B. cinerea*. Compared to control infected plants, data showed a stronger accumulation of transcripts encoding *PR1*, *PAD3*, *PR2*, and *plant defensin 1.2* (*PDF1.2*) mainly at 48 h post-inoculation (hpi; Figures 4C,D).

### Xh-Induced Resistance to *B. cinerea* Infection Is Dependent on Phytoalexin, SA, JA, and Ethylene Pathways

**FIGURE 5:**
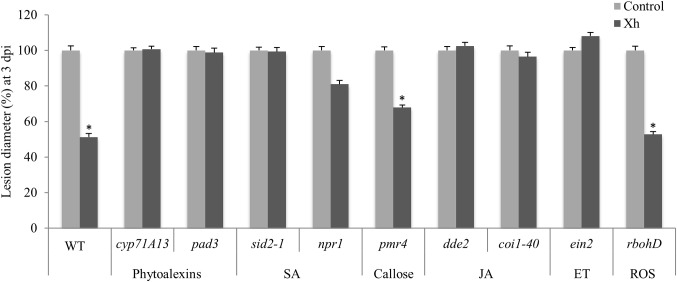
Characterization of defense pathways triggered during Xh-induced resistance against *B. cinerea* using Arabidopsis mutants. Two days after treatment with Xh (2.5 mg/ml) or water (control), mutants disrupted in the production of phytoalexin (*cyp71A13*, *pad3*), salicylate (*sid2-1*, *npr1*), jasmonate (*dde2*, *coi1-40*), ethylene (*ein2*), callose (*pmr4*), or reactive oxygen species (*rbohD*) were inoculated with *B. cinerea* (5.10^4^ conidia ml^-1^) and disease symptoms were measured at 3 dpi. Histograms represent the mean lesion diameter ± SE of three independent experiments (*n* = 60). Data are expressed as percentage of the water-treated control, set as 100%. Asterisks indicate statistically significant differences between control and elicitor-treated plants using a Student’s *t*-test (*P* < 0.05).

To determine the different pathways involved in this Xh-induced resistance, we used Arabidopsis deficient mutants. The *cyp71A13* mutant is missing the CYP71A13 enzyme that catalyzes the conversion of indole acetaldoxime to indole-3-acetonitrile in the camalexin synthetic pathway (Nafisi et al., 2007). The *pad3* mutant is unable to synthesize the cytochrome P450 catalyzing the final step in camalexin biosynthesis (Schuhegger et al., 2006). In these two mutants deficient in camalexin synthesis (*cyp71A13* and *pad3*), Xh was unable to induce resistance as this treatment did not significantly decrease the *B. cinerea* lesion size that is normally observed in WT plants (Figure 5). We also used SA-disrupted mutants such as *sid2-1*, blocked in the isochorismate dependent-SA biosynthetic pathway (Nawrath and Métraux, 1999) and *npr1*, unable to bind SA (Wu et al., 2012). Our data showed that the Xh-induced resistance was not active in these two Arabidopsis deficient mutants of the SA pathway (*sid2-1* and *npr1*; Figure 5). The *dde-2* mutant is defective in the *Allene Oxide Synthase* (*AOS*) gene encoding one of the key enzymes of the jasmonic acid biosynthesis pathway (von Malek et al., 2002) whereas *coi1-40* is deficient in the JA co-receptor Coronatine-Insensitive 1 (COI1) required for the known responses to this hormone (Katsir et al., 2008). Xh pretreatment on infected JA-deficient mutants *dde-2* and *coi1-40* did not significantly reduce the *B. cinerea* lesion diameter (Figure 5) demonstrating the essential role of the JA pathway in the Xh-triggered immunity against *B. cinerea*. We then investigated the role of the ethylene pathway in Xh-induced resistance against this pathogen. EIN2 is a key component in the ethylene signaling pathway, as demonstrated by completely ethylene-insensitive phenotypes of the *ein2* null mutants (Li et al., 2015). Xh treatment did not reduce the *B. cinerea* lesion size in this ethylene mutant (*ein2*; Figure 5). All these results suggest that camalexin, salicylic acid, jasmonic acid, and ethylene play a role in the Xh-induced resistance against *B. cinerea.* To further investigate the role of callose and ROS production in the Xh-triggered immune responses, we also performed protection assays in *pmr4* and in *rbohD*, a mutant impaired in the PAMP-triggered ROS production via the Respiratory Burst Oxidase Homolog D (Morales et al., 2016). Both *pmr4* and *rbohD* mutants conserved the Xh-induced resistance against *B. cinerea* (Figure 5) indicating that PMR4- and RBOHD-dependent responses are not required for Xh-induced resistance against *B. cinerea*.

## Discussion

The xyloglucans used in this study (Xh) are ß-1,4-glucan polymers associated with xylosyl, galactosyl, and fucosyl-type branching. A putative additional arabinosyl-type branching might exist in a minor structure of Xh as it has been previously described in sycamore (Kiefer et al., 1990) or different monocotyledons (Hsieh and Harris, 2009). Another possibility is that a putative 4-epimerase might transform a D-xylose in an L-arabinose during the apple fruit maturation, leading to an alternative structure of an oligomer possessing the same molecular weight (xylose and arabinose are two aldopentoses with the same molecular weight of 150.13). If the structures of the three Xh oligomers of DP 7, 8, and 9 have been clearly identified as XFGol, LFGol, and GLFGol, further H^1^-NMR investigations would be needed to precisely define the location of arabinose in a probable minor Xh component.

This study clearly demonstrates that Xh triggered a broad range of defense responses in grapevine and Arabidopsis (Figures 2, 3). Xh induce MAPK activation but do not elicit ROS production on Arabidopsis like cellobiose, a β-1,4-diglucose derived from cellulose (Souza et al., 2017). At the opposite, cellodextrins, which are longer derivatives of cellulose composed of linear β-1,4-glucosides, elicit an oxidative burst with a maximal H_2_O_2_ production triggered by the DP7 oligomer (Aziz et al., 2007). Treatment with Xh also triggers an increased expression of defense genes in both species, including *PAL* and *STS* in grapevine and *PAD3* and *PR1* in Arabidopsis. Upregulation of *STS* and *PAL* genes in grapevine and *PAD3* in Arabidopsis was also observed upon OG treatment with similar kinetics (Aziz et al., 2004; Denoux et al., 2008; Manzoor et al., 2013). By contrast, *PR1* expression revealed time point differences between Xh and OG treatments. In Arabidopsis, *PR1* transcripts accumulated within 1 h upon OG treatment before decreasing at 3 h (Manzoor et al., 2013) whereas Xh treatment induced an accumulation of *PR1* transcripts at 8 h, peaking at 24 h before declining at 48 h (Figure 3C). Moreover, Xh treatment elicits an accumulation of resveratrol (Figure 2E), the main phytoalexin produced by grapevine *via* the phenylpropanoid pathway in response to microbial attacks. These results are consistent with the induced *PAL* and *STS* gene expression (Figures 2C,D). Resveratrol is found to be involved in grapevine resistance against some pathogens including *B. cinerea* or *Plasmopara viticola* (Hasan and Bae, 2017). However, resveratrol accumulated in higher amounts in the medium of OG-treated cells compared to Xh-treated cells suggesting that Xh is an oligosaccharide possessing a lower elicitor activity. Indeed, even though the Xh are able to activate multiple defense related-pathways participating in PTI (Bigeard et al., 2015), they elicit more transient immune responses (MAPK activation, expression of defense genes, phytoalexin production) compared to other molecular patterns such as OG, laminarin, or flg22, previously described as active elicitors in grapevine (Aziz et al., 2003; Poinssot et al., 2003; Trdá et al., 2014). Interestingly, the xyloglucan nonasaccharide XXFG, produced by enzymatic hydrolysis of natural xyloglucans, was also found to promote the accumulation of phytoalexins in soybean and to induce resistance of wheat kernels against *Fusarium culmorum* (Pavlova et al., 1996). The fact that cellobiose, cellodextrins, and xyloglucans are able to trigger plant immune responses in different plant species suggest that a common β-1,4-glucan backbone is probably the basal structure of these DAMPs, even if specific DP and carbohydrate decorations might enhance their elicitor activity.

Previous studies have shown that the well-characterized OG protect Arabidopsis and grapevine against the necrotrophic fungus *B. cinerea* (Aziz et al., 2004; Ferrari et al., 2007). Surprisingly, our data show that OG did not induce a significant resistance in grapevine leaf disks against *B. cinerea* (Figure 2F). However, this discrepancy might be explained by a different level of virulence depending on the *B. cinerea* isolate or the plant genotype used, but also on whether leaves were wounded or not before inoculation (Vallejo et al., 2003; Kliebenstein et al., 2005). Interestingly, we demonstrated that the Xh enhance resistance against *B. cinerea* on grapevine and Arabidopsis plants. We also showed an Xh-induced resistance against the biotrophic oomycete *H. arabidopsidis* in Arabidopsis (Supplementary Figure S5). Together, all these results suggest that the hemicellulose-derived Xh are new elicitors that trigger immune responses and enhance resistance against necrotrophic and biotrophic pathogens in different plant species. The fact that the Xh induce multiple immune responses and trigger protection against biotrophic and necrotrophic pathogens might lead to interesting applications to limit the use of chemicals to protect plants.

To further investigate the molecular components involved in xyloglucan signaling, we used Arabidopsis mutants. We confirmed that the two MAPKs phosphorylated upon Xh treatment were MPK3 and MPK6 (Figure 3A). These two MAPKs are activated in response to pathogens and elicitors such as OG or flg22 (Galletti et al., 2011). Callose deposition is typically triggered by conserved PAMPs and DAMPs such as flg22, chitosan, or OG (Luna et al., 2011; Ferrari et al., 2013). Here, we demonstrated that Xh trigger callose deposition after *B. cinerea* infection (Figure 4A). Using the *pmr4* mutant, our results indicated that the Xh-induced callose production is dependent on PMR4 (Figure 4B), the flg22-induced callose synthase (Luna et al., 2011). However, Xh-induced callose deposition is not an essential defense response upon *B. cinerea* infection in Arabidopsis since the *pmr4* mutant is still protected by Xh (Figure 5). In the same way, Xh-induced activation of defense responses effective against *B. cinerea* does not require *AtrbohD*, as we did not detect significant differences in Xh-induced resistance between WT and *AtrbohD* plants (Figure 5). Similarly, OG treatment of the *pmr4* and *AtrbohD* mutants resulted in a conserved protection against *B. cinerea*, indicating that callose and H_2_O_2_ does not play a major role in OG-induced resistance against this pathogen (Galletti et al., 2008). It is well established in the literature that the ethylene (ET) and jasmonic acid (JA) pathways take part in basal defense against necrotrophic pathogens and control the expression of some *PR* genes such as the plant defensin *PDF1.2* (Glazebrook, 2005). Mutations that block JA or ET signaling, including *coi1* and *ein2*, cause enhanced susceptibility against *B. cinerea* (Thomma et al., 1998, 1999; Ferrari et al., 2003). We have shown that Xh-induced resistance against *B. cinerea* is dependent on the JA and ET pathway as *coi1*, *dde2*, and *ein2* mutants did not show any more Xh-triggered immunity (Figure 5). In Arabidopsis, resistance to *B. cinerea* was also found to involve the phytoalexin camalexin as *pad3* mutant showed higher susceptibility to the fungus and camalexin was found to have a toxic effect against *B. cinerea* (Ferrari et al., 2003; Kliebenstein et al., 2005). In this study, the Xh were unable to induce protection in *cyp71A13* and *pad3* Arabidopsis mutants, showing that camalexin plays a part in Xh-induced resistance against *B. cinerea* (Figure 5). Last, our data indicate that Xh-induced resistance seems to be dependent on SA signaling as *sid2-1* and *npr1* did not show Xh-induced resistance (Figure 5). This result might be surprising because SA-dependent responses are not predicted to play a major role in resistance against necrotrophic pathogens (Pieterse et al., 2012). However, plant hormone signaling pathways are interconnected in a complex network and there are some evidences that SA might contribute to resistance against *B. cinerea*. In particular, SA-mediated responses appear to be important in limiting the fungal spread at the site of infection in Arabidopsis (Govrin and Levine, 2002; Ferrari et al., 2003), tomato (Angulo et al., 2015), or grapevine (Kelloniemi et al., 2015). Strikingly, OG and flg22 were shown to induce resistance against *B. cinerea* independently of SA, JA, and ET pathways (Ferrari et al., 2007), suggesting that the Xh act through a divergent signaling pathway to rapidly counteract attack by pathogens. Spatiotemporal kinetics of hormone biosynthesis and signaling during the plant/pathogen interaction is important (Koornneef et al., 2008; Leon-Reyes et al., 2010). Our results suggest that Xh directly elicit the expression of *PAD3*, involved in the camalexin biosynthesis, and of the SA-marker gene *PR1* before triggering during *B. cinerea* infection callose deposition and the overexpression of *PDF1.2*, a marker gene of the JA/ET-dependent pathway.

The activation of Xh-triggered immune responses in grapevine and Arabidopsis suggests that these plant species perceive hemicellulose derived-xyloglucans and thus possess at least a cognate receptor. To further investigate how Xh are perceived, it would be therefore necessary to identify the PRRs involved in the recognition and signaling triggered by Xh. Recent studies have revealed that BAK1, CERK1, and SOBIR1 are frequently involved in signal transduction following the perception of different PAMPs including flg22, chito-oligosaccharides, and the elongation factor EF-Tu (Heese et al., 2007; Miya et al., 2007; Wan et al., 2008; Brutus et al., 2010; Roux et al., 2011; Boutrot and Zipfel, 2017). Thus, it would be interesting to check if these co-receptors play a role in the Xh-triggered signaling.

More globally, we can conclude that cell-wall fragments of pectins (OG), cellulose (cellobiose and cellodextrins), and hemicellulose (xyloglucans) should be all considered as DAMPs which are able to trigger the plant immunity. They can be released by cell wall degrading enzymes (CWDEs) produced by pathogens such as PGs, cellobiohydrolases, endoglucanases, xyloglucanases, and often referred as carbohydrate-active enzymes (or CAZymes). For instance, the genome of the necrotrophic fungus *B. cinerea* contains 367 genes encoding putative CAZymes including some well-known CWDEs (Amselem et al., 2011). Nevertheless, addition of Xh in the culture medium did not increase its growth *in vitro* (Supplementary Figure S3) suggesting that CWDEs were already present to degrade carbohydrates of the potato dextrose broth medium. Once produced these active oligosaccharide fragments elicit plant immune responses and then need to be degraded. Recently, Benedetti et al. (2018) identified four Arabidopsis berberine bridge enzyme-like proteins which are OG oxidases that reduced the elicitor activity of OG with a DP > 4. Similarly, different glycoside hydrolases and glycosyltransferases from Arabidopsis and tomato have been found to modify the carbohydrate composition of the xyloglucans leading to their putative degradation and to the existence of an important structural biodiversity in these cell wall oligomers (Schultink et al., 2014). Thus, it would be interesting to study the biological immune responses triggered by xyloglucans possessing different carbohydrate decorations to find the most active epitopes.

All the xyloglucans used in this study have been reduced by NaBH_4_ to facilitate the purification process and the NMR analysis and thus possess a glucitol (sorbitol) instead of a glucose at the reducing end. It would be also interesting to compare the elicitor activity of the native and reduced xyloglucans. Indeed, chemical or enzymatic modifications of the reducing end in OG oligomers were shown to alter their biological activity (Spiro et al., 1998; Benedetti et al., 2018). Particularly the chemical reduction of OG DP13 by NaBH_4_ or tyramination of its reducing end decreased its ability to elicit the extracellular pH alkalinization and the H_2_O_2_ production in tobacco cell suspensions (Spiro et al., 1998). This might explain why we should use a rather high concentration of Xh (at the mM range) to detect an elicitor activity in grapevine or Arabidopsis. Thus, further experiments will be needed to investigate the perception of the different xyloglucans and the mechanisms underlying the specificity of the Xh-triggered immune responses in different plant species.

## Materials and Methods

### Plant and Cell Culture Materials

Grapevine (*V. vinifera* cv. Marselan) herbaceous cuttings were grown in individual pots (10 cm × 10 cm × 7 cm) containing a mixture of blond peat and perlite (3/2, v/v) in a greenhouse [24 and 18°C, day and night, respectively, relative humidity (RH) 50 ± 10%, and a 16 h light period], until they developed six leaves (ca 7 weeks later). Plants were watered with a fertilizer solution (NPK 10-10-10, Plantin, France). Grapevine cells (*V. vinifera* cv. Gamay) were cultivated and collected as described previously (Trdá et al., 2014).

Arabidopsis (*A. thaliana*) seeds of the WT ecotype Columbia (Col-0) and mutants in the same background were obtained from the Nottingham *Arabidopsis* Stock Center (NASC). Plants were grown under a 10/14 h day/night cycle at 20/18°C, respectively, with a light intensity of 175 mmol m^-2^ s^-1^ provided by fluorescent tubes.

Arabidopsis (Col-0) cells were cultivated as described previously (Trdá et al., 2014). Cell suspensions were collected during the exponential growth phase and washed by filtration in a suspension buffer containing 175 mM mannitol, 0.5 mM K_2_SO_4_, 0.5 mM CaCl_2_, and 2 mM MES adjusted to pH 5.3. Cells were resuspended at 0.1 g.ml^-1^ fresh weight (FW). After 1 h of equilibration (130 rpm, 25°C), cells were treated with elicitors and analyses were performed.

### Elicitors

Purified xyloglucans (Xh) and OG were provided by Elicityl (Crolles, France^1^). The purified Xh possess a DP of 7-9 (product GLU1111) and OG have a mixed average DP of 10/15 (product GAT114). The elicitors were prepared in ultra-pure water and tested for their non-toxicity 24 h after treatment on *Arabidopsis* cell suspensions. Xh and OG were used at 1 mg/ml for all experiments, except for Arabidopsis gene expression, callose production, and protection assays realized at 2.5 and 5 mg/ml for grapevine protection assays. Acid hydrolysis of Xh was performed by treatment with 2 M trifluoroacetic acid during 4 h at 100°C. The reaction was then stopped by neutralization with 2 M NaOH and the hydrolyzed Xh (Xh_H) preparation was desalted by chromatography. Finally, neutral pH was checked and the solution was filtered through 0.2 μm before application on plants.

### H_2_O_2_ Detection

In Arabidopsis and grapevine cell suspensions, H_2_O_2_ production was detected using the chemiluminescence of luminol, as described in Dubreuil-Maurizi et al. (2011).

### Immunodetection of Phosphorylated MAPKs

Twenty micrograms of protein per sample were solubilized in Laemmli buffer (Laemmli, 1970), submitted to 12% SDS-PAGE before Western blotting. After transfer, the nitrocellulose membrane (Hybond ECL, Amersham Biosciences, Munchen, Germany) was pre-incubated first during 1 h at room temperature with TBST buffer (10 mM Tris-HCl, 150 mM NaCl, 0.05% Tween-20, pH 7.5) and 2% BSA, then incubated for 1 h with an anti-phospho Thr202/Tyr204 peptide of human ERK1/2 mouse antibody (Cell Signaling, Danvers, MA, United States), 1/20,000 diluted in TBST buffer. After three washes with TBST buffer, probing and detection were performed by an ECL detection kit (Perkin Elmer, Little Chalfont, United Kingdom).

### Protection Assays

First, 3-week-old Arabidopsis plants were sprayed with elicitors (2.5 mg/ml). Two days after treatment, *B. cinerea* infections were performed according to Manzoor et al. (2013). For *B. cinerea* infections on grapevine, leaf disks were incubated for 48 h on aqueous solutions containing Xh or OG (5 mg/ml), then inoculated with *B. cinerea* conidial suspension of 5.10^4^ conidia/ml. Quantification of disease development in Arabidopsis and grapevine leaves after inoculation was measured as average diameter of lesions formed during infection.

For *Hyaloperonospora arabidopsidis*, NOCO_2_ strain was spray-inoculated to saturation with freshly harvested spores (5.10^4^ spores.ml^-1^). Plants were kept in a growth chamber under high humidity. The seventh day, aerial parts of plants were harvested, pooled for each treatment, and weighed. The liberated spores were counted under microscope and infection intensity was calculated as number of spores.g^-1^ of plant FW.

### Gene Expression Analyses

For Arabidopsis, three leaves from three independent plants were collected and frozen in liquid nitrogen at the different time points. For grapevine, 2 ml aliquots of treated cells were collected at 0, 1, 3, 6, 9, and 24 h post treatment and frozen. Total RNA was extracted using TRIzol reagent, following the manufacturer’s protocol (Invitrogen, Carlsbad, CA, United States). Five hundred nanograms of total RNA was reverse transcribed using the M-MLV Reverse Transcriptase kit (Promega, Madison, WI, United States) for Arabidopsis and 1 μg of total RNA using Superscript III reverse transcriptase kit (ThermoFisher, Waltham, MA, United States) for grapevine. Real-time quantitative (q)PCR was performed using 5 ng/μl cDNA, qPCR SYBR green ROX mix (containing Taq polymerase, deoxyribonucleotide triphosphate, and SYBR green dye; Thermo Scientific, Waltham, MA, United States) and 200 nM primers (Table 1) in a 5-μl volume. Triplicate quantitative assays per biological experiment were performed by using the ViiA^TM^ detection system (Life Technologies, Carlsbad, CA, United States). The activation factor of the relative gene expression was determined with the comparative cycle threshold (Ct) method 2^-ΔΔ*Ct*^ (Livak and Schmittgen, 2001). The Arabidopsis housekeeping *OLI* gene (*At4g26410*) and the grapevine housekeeping gene *EF1*α (*XM_002284888.1*) were used as internal controls for normalization (Czechowski et al., 2005; Dubreuil-Maurizi et al., 2010).

**Table 1:** Sequences of primers used for real-time quantitative polymerase chain reaction (qPCR).

Names	Primers
*PAL*	Forward: 5′-AGTCTCCATGGACAACACCCG-3′
	Reverse: 5′-TGCTCAGCACTTTCGACATGG-3′
*STS*	Forward: 5′-TACGCCAAGAGATTATCACT-3′
	Reverse: 5′-CTAAAGAGTCCAAAGCATCT-3′
*PR1*	Forward: 5′-ACTACAACTACGCTGCGAACA-3′
	Reverse: 5′-TGGCTTCTCGTTCACATAATTCCC-3′
*PR2*	Forward: 5′-GGGACGGCTCTCGTGGCTACC-3′
	Reverse: 5′-CGCGCGTTATCGAAACTCGCGG-3′
*PAD3*	Forward: 5′-GGGTACCATACTTGTTGAGATGG-3′
	Reverse: 5′-TTGATGATCTCTTTGGCTTCC-3′
*PDF1.2*	Forward: 5′-CACCCTTATCTTCGCTGCTCTTG-3′
	Reverse: 5′-CACTTGTGTGCTGGGAAGACATAG-3′
*OLI*	Forward: 5′-GAGCTGAAGTGGCTTCCATGA-3′
	Reverse: 5′-GGTCCGACATACCCATGATCC-3′
*EF1α*	Forward: 5′-GAACTGGGTGCTTGATAGGC-3′
	Reverse: 5′-AACCAAAATATCCGGAGTAAAAGA-3′


### Resveratrol Quantification in *Vitis vinifera*

At different times after treatment, cell suspension aliquots (2 ml) were collected and filtered on GF/A filters to quantify the resveratrol in the culture medium, as previously described (Lachhab et al., 2014). Filtrates were directly analyzed by ultra HPLC (Waters, Milford, MA, United States) following the method described by Boutegrabet et al. (2011). Each sample (10 μl) was loaded onto a BEH C-18 column (Waters, Eschborn, Germany) equilibrated with water–acetronitrile–formic acid (100:10:0.1) and acetonitrile. Resveratrol was eluted with a linear gradient from 0 to 42% acetonitrile at a flow rate of 0.65 ml/min. Quantification of resveratrol was performed with standard calibration curves, using peak areas of different amounts of pure molecules, fluorometrically detected (λex = 330 nm, λem = 400 nm) and the chromatographic characteristics were calculated using Waters Empower software.

### Callose Deposition Detection

Callose deposition was revealed by aniline blue staining as described by Gauthier et al. (2014). Briefly, clarified leaves were stained in 0.05% aniline blue (in 0.1 M Na_2_HPO_4_ phosphate buffer, pH 8) overnight and then mounted on microscope slides in the same solution. Callose deposition was observed in blue by epifluorescence microscopy under UV (λex = 340 nm; λem = 380 nm, stop filter LP 430 nm, Leica). Relative callose deposition was quantified as the number of fluorescent callose-corresponding pixels relative to the total number of pixels covering plant material, using “Shanbhag” automatic thresholding of the ImageJ software. Average callose measurement was quantified at 3 dpi on five images per infection site from six leaves coming from three independent plants per modality.

### Test of Xyloglucan Toxicity on *Botrytis cinerea* Growth

*Botrytis cinerea* conidia (2.10^5^/ml) were prepared in Potato Dextrose Broth (6 g/l, Difco, United States). The suspension of *B. cinerea* was mixed with Xh solution, beforehand sterilized with Millex 0.22 μm sterile filter. Triplicates of 300 μl (54.10^3^ conidia/well) of this mixture were dispatched in a 100-well microplate. *B. cinerea* mycelial growth was estimated by automatic optical density measurements at 492 nm and 25°C by a microplate reader (Bioscreener) every 2 h and until 66 h.

## Author Contributions

JC, SB, and CL-G performed most of the experiments. CL-G, XD, and BP conceived the original screening and research plans. DB, AC, MCH, and BD provided technical assistance. MCH, XD, and BP supervised the experiments. JC, SB, EN, LG, XD, MCH, and BP designed the experiments and analyzed the data. JC, XD, BD, and BP conceived the project and wrote the article with contributions of all the authors.

## Conflict of Interest Statement

The authors declare that the research was conducted in the absence of any commercial or financial relationships that could be construed as a potential conflict of interest.

## Footnotes

1 http://www.elicityl-oligotech.com/
