# Supplementary material for: The Cell Wall-Derived Xyloglucan Is a New DAMP Triggering Plant Immunity in Vitis vinifera and Arabidopsis thaliana
Source: Front Plant Sci. 2018 Nov 28;9:1725. doi: 10.3389/fpls.2018.01725 (PMC6280107; doi:10.3389/fpls.2018.01725)
Supplement: Supplementary file 1 [file Data_Sheet_1.PDF]

A

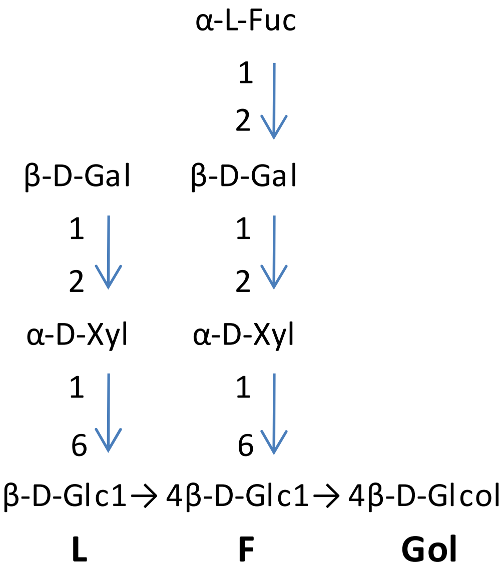

B

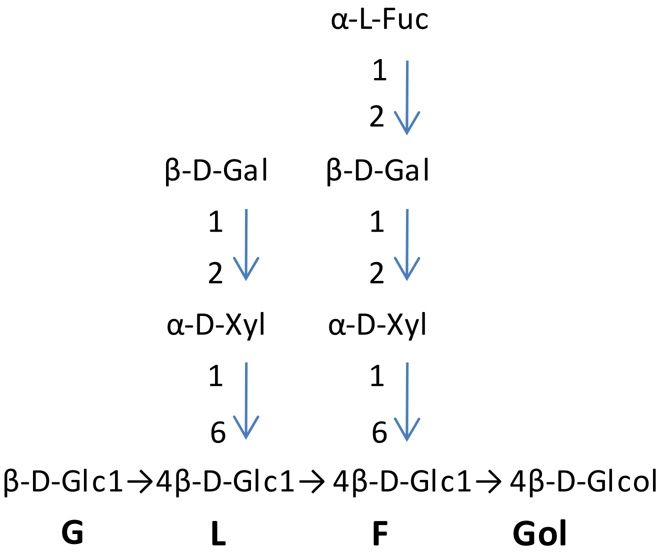

C

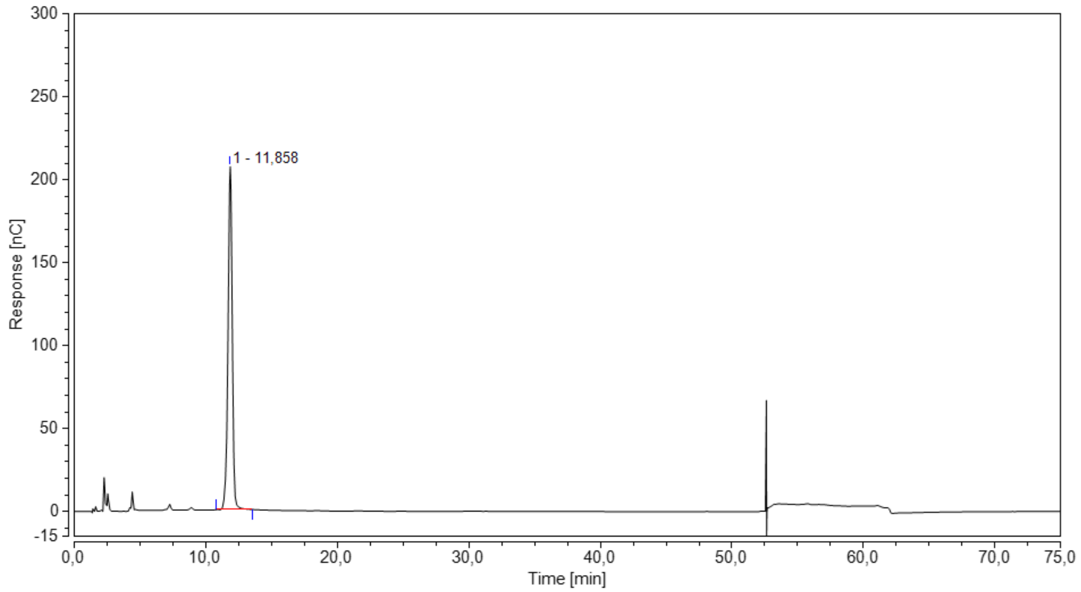

Figure S1. Structures of the minor components of the purified xyloglucan.
A. Structure of the purified xyloglucan DP8 (LFGol; retention time: 33.9 min, 8.0% AUC) according to the oligosaccharide nomenclature described by Fry *et al*. (1993). **B.** Structure of the purified xyloglucan DP9 (GLFGol; retention time 34.5 min, 3.6% AUC) according to the oligosaccharide nomenclature described by Fry *et al*. (1993). **C**. HPAEC-PAD PA100 chromatogram of a cellobiose standard showing a retention time of 11,86 min. Injection has been performed at 50 mg/L.

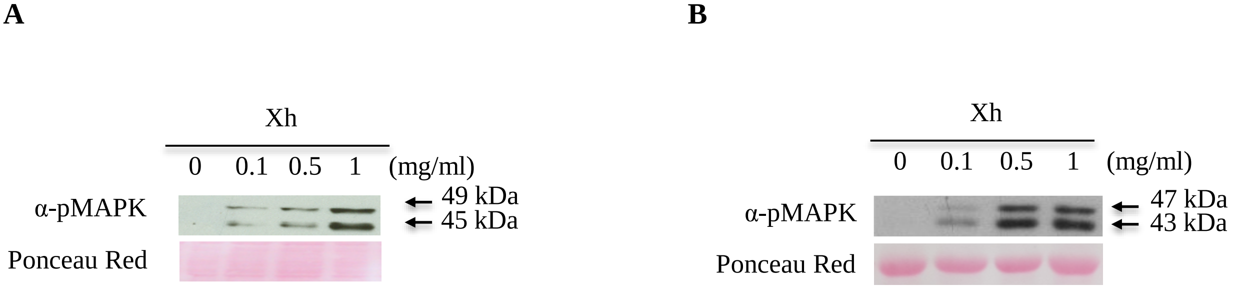

Figure S2. MAPKs phosphorylation induced by Xh in grapevine cells (A) and Arabidopsis plants (B).
A. Dose-dependent Xh–induced MAPK activation in grapevine (*V. vinifera*) cells at 10 min after treatment at different concentration (0.1, 0.5 and 1 mg/ml) detected by immunoblotting with α-pERK1/2. Equal protein loading was checked by Ponceau red staining. Results are from one representative experiment out of two. **B.** Dose-dependent Xh–induced MAPK activation in Arabidopsis plants at 10 min after treatment at different concentrations (0.1, 0.5 and 1 mg/ml) detected by immunoblotting with α-pERK1/2. Equal protein loading was checked by Ponceau red staining. Results are from one representative experiment out of two.

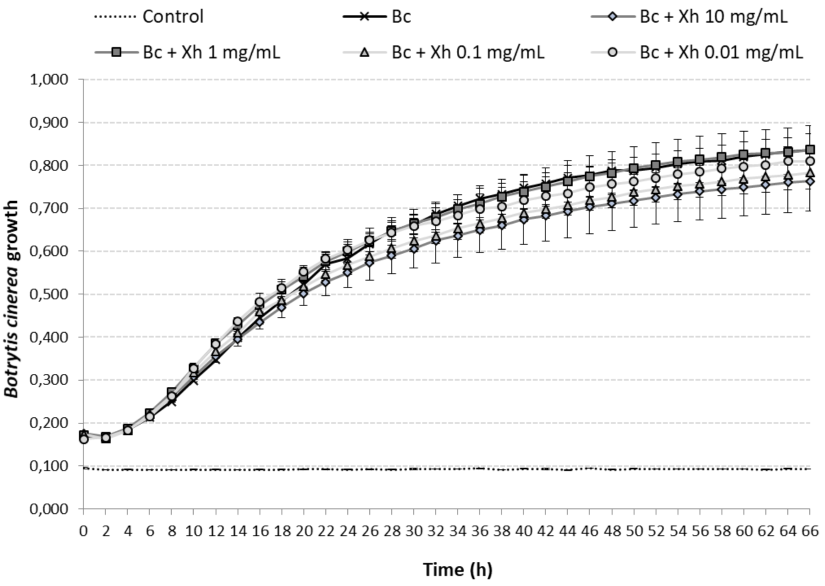

Figure S3. **Toxicity assay of Xh on the *B. cinerea* growth *in vitro*.**

B. cinerea conidia (2.10^5 conidia/ml) were untreated (Bc) or treated *in vitro* with Xh at 10 mg/ml, 1 mg/ml, 0.1 mg/ml, 0.01 mg/ml and mycelial growth was measured by optical density at 492 nm (microplate reader Bioscreener) during 66 h of treatment. Control corresponds to Xh at 10 mg/ml without *B. cinerea* conidia. Data represent the mean ± SD of triplicate assays. Results are representative of 3 independent experiments. No significant difference found between untreated *B. cinerea* vs *B. cinerea* treated with Xh according to Student's *t*-test ($P< 0.05$).

A

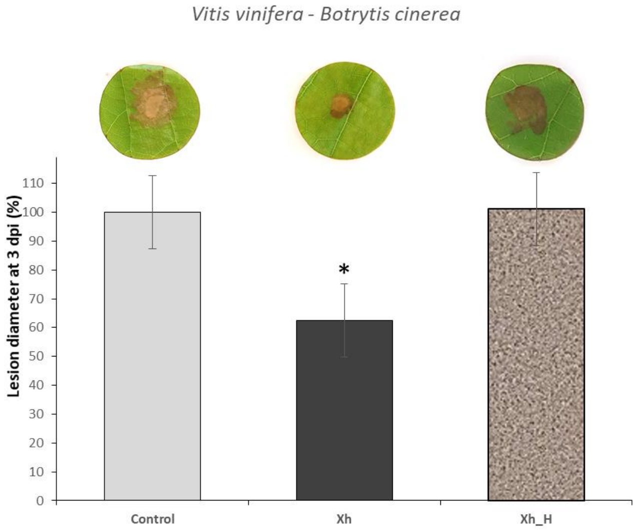

B

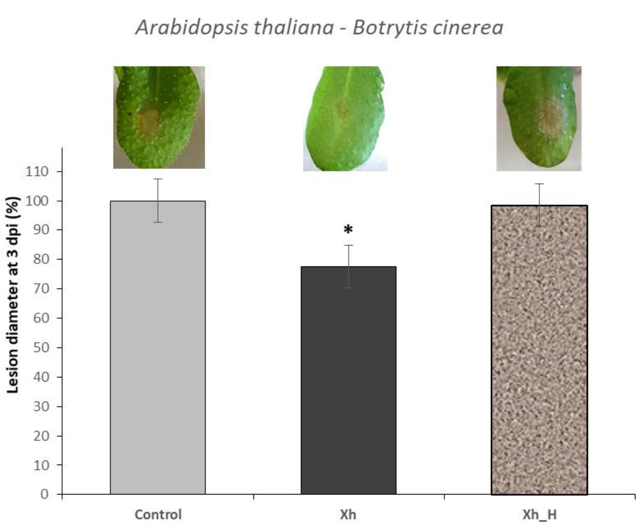

Figure S4. Loss of induced resistance in grapevine and Arabidopsis when the Xh undergo acid hydrolysis
Xh was submitted to an acid hydrolysis and induced resistance against *B. cinerea* was tested in grapevine (**A**) or Arabidopsis (**B**). **A.** *V. vinifera* leaf disks were incubated for 48 h on aqueous solutions containing water (control), Xh (5 mg/ml) or hydrolysed Xh (Xh_H, 5 mg/ml) before inoculation with a 6-µL droplet of a conidial suspension (5.10^4 conidia/ml) of *B. cinerea* (40 disks per condition). Disease assessment was determined measuring the average diameter of lesions formed 3 days post inoculation (dpi) and reported to the water control, set as 100%. **B.** Two days after treatment by spraying with water (control), Xh (2.5 mg/ml) or hydrolysed Xh (Xh_H, 2.5 mg/ml), *A. thaliana* Col-0 plants were inoculated with *B. cinerea* (6-µL droplet at 5.10^4 conidia ml^{-1}) and disease symptoms were measured at 3 dpi and reported to the water control, set as 100%. Data represent the average diameter of lesions ± SD (40 inoculation sites per condition). Similar results were obtained in at least two independent biological experiments for each plant species. Asterisks indicate significantly different values between treated vs control plants according to Student's *t*-test (P< 0.05). A representative image for each modality is shown.

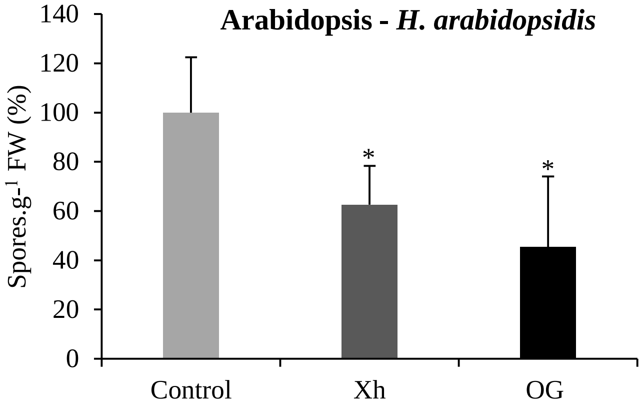

Figure S5. **Xh-induced resistance in Arabidopsis against *Hyaloperonospora arabidopsidis*.**
Two-week-old Col-0 plants were treated with water (control), Xh (2.5 mg/ml) or OG (2.5 mg/ml) then, 48 h later, plants were sprayed with *H. arabidopsidis* spores (5.10^4 spores.ml^{-1}). Data represent the percentage of spores.g^{-1} of plant fresh weight (FW) in elicitor-treated plants compared to control-treated plants (set as 100%) at 7 dpi. The histogram indicates means ± SD of three independent experiments. Asterisks indicate statistically significant differences between control and elicitor-treated plants using a Student's *t*-test (P<0.05).

	Expected quantities (mol)	(%DW)	Observed quantities (%DW)
Fucose	1	13.5	12.6
Galactose	1	15.0	15.1
Glucose	2	30.1	23.6
Xylose	2	24.5	18.0
Glucitol	1	16.9	14.2
Arabinose	0	0.0	2.3

Table S1. Expected and observed relative amounts of each monosaccharide after acid hydrolysis of Xh. Percentages of observed dry weight (DW) have been determined based on the analysis of the HPAEC-PAD PA1 chromatogram presented in Fig. 1A. The expected quantities are based on the theoretical relative amount of each monosaccharide in the heptamaloxyloglucan (Xh DP7) described in Fig. 1E.
